# Baseline CD4 count and the time interval between the initial HIV infection and diagnosis among PLHIV in Bhutan

**DOI:** 10.1002/iid3.444

**Published:** 2021-05-04

**Authors:** Lekey khandu, Guru P. Dhakal, Karma Lhazeen

**Affiliations:** ^1^ Communicable Disease Division, Department of Public Health Ministry of Health Thimphu Bhutan; ^2^ Department of Medicine Jigme Dorji Wangchuck National Referral Hospital Thimphu Bhutan; ^3^ Department of Public Health Ministry of Health Thimphu Bhutan

**Keywords:** AIDS viruses, CD4 cell count, CD4 counts, CD4 lymphocyte counts, human immunodeficiency virus, T4 lymphocyte count

## Abstract

**Introduction:**

CD4 count is an important predictor of disease progression, opportunities infection, deaths, and to understand the time interval between initial HIV infection to the first diagnosis. However, baseline CD4 count and the time period between initial infection and the diagnosis amongst PLHIV in Bhutan never been evaluated.

**Methods:**

This is a retrospective study of the diagnosed PLHIV from the existing data system from January 10 to 30, 2021. Out of 512 reported HIV cases, 488 of those who were more than or equal to 18 years old and had their CD4 count testing within 6 months before initiating ART were considered for analysis. Descriptive statistical analysis was used to analyze the characteristics of the study population and relationship were established using the *χ* ^2^ Test. We have sought ethics approval and waiver for informed consent as it is the retrospective study of the client's record. The client's confidentiality was ensured by removing all the identifiers.

**Results:**

The mean CD4 was 345 cells/ml for males and females. Twenty‐five percent of the clients had CD4 counts below 200, 30%, between 200 and 349, 25% between 350 and 499, and 20% above 500 cells/ml. A larger number of males showed a CD4 count below 200 cells/ml while more females showed a CD4 count more than 500 cells/ml. The mean time interval between initial infection to the first diagnosis was 4 years in males and females. However, about one‐fourth were found to have been infected between 5 and 8 years before diagnosis and less than 10% were diagnosed within less than 1 year of infection.

**Conclusions:**

The study revealed a late diagnosis of HIV infection in Bhutan thereby risking the transmission to the community and risk of severe disease and mortality. The upscaling of voluntary counseling and testing, medical screening, and alternative methods like community‐based testing including HIV Self Testing for early detection needs to be implemented in the country.

## Graphical abstract

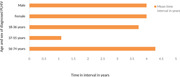

## 1 INTRODUCTION

Bhutan reported the first HIV‐positive case in 1993. As of December 2020, of the estimated 1300 HIV cases in the country a cumulative of 741 (52% male and 48% female) have been diagnosed. Of these, 557 are alive and 539 of them are on antiretroviral (ARV) treatment.^1^ Until today, CD4 count has remained as one of the most important means in managing PLHIV. The evidence shows that CD4 count is a vital predictor of disease progression and deaths.^2^, ^3^, ^4^, ^5^ The baseline CD4 count has always been considered for the initiation of ART among the PLHIV until the treat all policy was introduced. In 2005 Bhutan initiated ART at the CD4 count less than 200 cells/mm^3^ followed by less than or equal to 350 and less than or equal to 500 cells/mm^3^, all based on WHO guidelines.^6^ From 2016 the “treat all policy” was implemented wherein treatment was initiated irrespective of CD4 count.^6^ In Bhutan, before the era of treat, all policy CD4 count testing was carried out every 6 months. Now with the introduction of the policy and the availability of routine viral load testing, it is carried out for the newly diagnosed cases and on those who are lost to follow‐up clients re‐entering into the continuum of care.^7^

Although the viral load is considered a scientifically proven indicator to understand the treatment outcome and effectiveness of treatment regimens,^8^ CD4 cell count helps in monitoring the risk of opportunistic infection (OI) and treatment failure.^9^ The studies have found that individuals with higher baseline CD4 counts at the initiation of the ARV have the best chance for full immune reconstitution.^10^ Furthermore, the baseline CD4 count among the diagnosed PLHIV is important to determine the approximate time of HIV infection to understand whether there is a delay in case of diagnosis since infection and appropriate interventions can be taken to enhance early diagnosis.^11^ The delayed HIV diagnosis results in advanced immune compromise, increased risk of treatment failure,^12^ loss of opportunities for preventing onward transmission, and ultimately increases costs for the healthcare system.^13^, ^14^, ^15^ In many of the countries, HIV cases are diagnosed very late thus resulting in high morbidity and mortality. The evidence showed that the PLHIV diagnosed with CD4 less than 350 cells/mm^3^ has three times more likely to suffer deaths as compared to PLHIV with an early diagnosis that is CD4 count more than 350 cells/mm^3^.^16^

The past studies have also demonstrated that treatment outcome and CD4 cell recovery rate can be driven by the time at which care, support, and treatment is started.^17^, ^18^ Therefore, understanding the baseline CD4 count of the diagnosed treatment naïve PLHIV is very important. At present, there is no baseline CD4 count information of the diagnosed PLHIV in Bhutan, and therefore difficult to understand whether the cases are diagnosed late or the current HIV testing intervention is picking up the recent new infections. As such, this study will assess the baseline CD4 count and calculate the time delay by taking an average CD4 decay per calendar year among all 488 treatment‐naive diagnosed PLHIV.

## 2 METHODS

This is a retrospective study using the HIV‐positive client's records and was conducted from January 10 to 30, 2021. The study protocol was reviewed and approved by the Research Ethics Board of Health and upon the request of the study team, the informed consent was waived off owing to no more than minimal risk to the participants. The client's confidentiality was protected by removing the identifiers such as names, citizenship identity card numbers, and their addresses by the in‐charge of the database and a duplicate excel of was created with no identifiers for the study team. These records were kept in locked cabinets of the HIV care and treatment unit.

### 2.1 Study setting

The study was conducted at the Care, Support, and Treatment (CST) unit, Jigme Dorji Wangchuck National Referral Hospital (JDWNRH), Bhutan. The CST unit is the nodal agency in managing all the diagnosed PLHIV in the country. Bhutan is a landlocked country in South‐East Asia, located at the eastern end of the Himalayas, with a total population of approximately 748,931.^19^ The country has 2187 allopathic (337 doctors, 1202 nurses, 44 pharmacists, and 604 health assistants) and 116 traditional healthcare providers.^20^

### 2.2 Subheading

#### 2.2.1 Study population and sampling

The study included a total of 488 HIV cases above 18 years old at the time of diagnosis from 1994 to December 2020. The convenient nonrandom sampling method was used to select the participants who had carried out their baseline CD4 count testing within 6 months before the initiation of the ART. Clients who did not have baseline CD4 counts were excluded from the study.

#### 2.2.2 Data collection tool

We have used the excel database maintained to manage the HIV cases in the country at the Care, Support and Treatment (CST) Unit of the National HIV, AIDS, and STIs Control Program (NACP) as detailed below under the data processing and analysis.

#### 2.2.3 Data processing and analysis

The data were extracted from the main excel database of the CST. All identifiers like name, addresses, phone number, and place of detection were removed by the concerned HIV/AIDS Counselor of the CST unit before giving access to the study team. The excel database with no identifiers was further cleaned by removing the unwanted variables like occupation, level of education, nationality, mode of transmission, mode of diagnosis, viral load, tuberculosis screening, and ART‐related information. The cleaned data from the excel sheet was then entered into the EpiData software for analysis. Descriptive statistical analysis was used to analyze the demographic characteristics of the study population and the relationship between variables was computed using the *χ* ^2^ Test. We have used the initial baseline CD4 count value as an indicator of the time delay between HIV infection and diagnosis based on the average CD4 decay per calendar. The time delay was calculated by taking an average CD4 decay per calendar year among all 488 PLHIV clients.^21^

The formula used to determine the approximate time when HIV was acquired based on CD4 values is *t* = *N* – *n*/80, where *t* is the approximate time of initial HIV infection, *n* is the mean CD4 count at initial diagnosis, 80 represents the approximate annual CD4 decay rate,^22^ and *N* is the average reference CD4 count among healthy Bhutanese blood donors, which is as 668.3 cells/μl for both males and females.^23^ We have not considered mean CD4 count by sex because the study revealed that although females have significantly higher counts (*p* = .004), these differences were numerically small.^23^ The categorization of the CD4 data was done according to the defined WHO criteria to initiate the ART based on the individual CD4 count values.^24^

## 3 RESULTS

We had a total of 488 clients (male 254 and female 234) who had undergone CD4 count testing within the 6 months before the initiation of the ART. The median age of the study population was 32 years (range, 18–74 years). The mean CD4 was 345 cells/ml for both males and females with a range of 2–1120 cells/ml. On the age distribution, the clients in the age range of 18–36 years had mean CD4 values of 345, between 37 and 55 years had 341 and 56–74 years had 326 cells/ml, respectively. On percentile of 0–25 percentile had 203 cells/ml, 25–50 percentile had 325, 50–75 percentile had 455, and 75–100 had 1120 cells/ml.

### 3.1 Categorization of CD4 count by WHO clinical staging

Table 1 shows the distribution of CD4 count by gender based on the WHO clinical staging. The study revealed that there were more females in WHO Stage 1 and Stage 3 disease and more males in Stage 2 and 4 diseases. The overall distribution of CD4 count revealed that the majority, 30% (*n* = 145) of the subjects had CD4 count between 200 and 349 cells/ml followed by 25% each for below 200 cells/ml and between 350 and 499, while 20% (*n* = 99) had above 500 cells/ml. A *χ* ^2^ test of independence showed that there was no significant association between gender and categories of CD4 count, *χ* ^2^ (3, *N* = 488 88) = 4.02, *p* = .258.

**Table 1:** Distribution of CD4 cell counts by sex and WHO clinical stage among the naive PLHIV in Bhutan

Clinical stages	CD4 cell counts in cells/ml	HIV associated symptoms	Male	Female	Row totals	%
1	CD4 count >500	Asymptomatic	48 (51.53)	51 (47.47)	99	20
2	CD4 count 350–499	Mild symptom	64 (64.02)	59 (58.98)	123	25
3	CD4 count 200–349	Advanced symptom	70 (75.47)	75 (69.53)	145	30
4	CD count <200	severe symptoms	72 (62.98)	49 (58.02)	121	25
	**Column totals**		**254**	**234**	**488** (**Total)**	**100**

### 3.2 CD4 distribution by age

**Figure 1:**
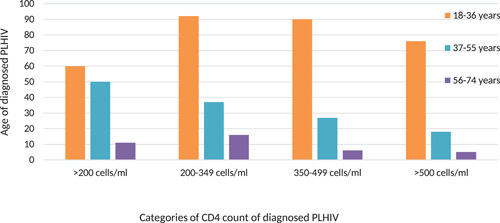
Distribution of CD4 count by age group among the naïve PLHIV in Bhutan

As indicated in Figure 1 across all categories of CD4 count the highest number of cases were in the age range of 18–36 years (65.16%, *n* = 318) followed by 37–55 years (27.04%, *n* = 132), and then in aged 56–74 years (7.78%, *n* = 38), respectively. The distribution of age for different categories revealed that for CD4 countless than 200 cells/ml, 12.29%, were between 18 and 36 years, 10.24% were between 37 and 55 years, and 2.25% were between 56 and 74 years. Similarly, for CD4 count between 200 and 499 cells about 37.29% (*n* = 182) were aged between 18 and 36, followed by 13.11% (*n* = 64) between 37 and 55 years and 4.50% (22) between 56 and 75 years. For CD4 count more than 500 cells about 77% were between 18 and 36 years, 18% between 37 and 55 years, and 5% between 56 and 74 years. Unlike other age categories the distribution of CD4 count among the population aged 37–55 years shows the decreasing trend with the highest number of PLHIV in CD4 cell range of less than 200 cells/ml.

**Figure 2:**
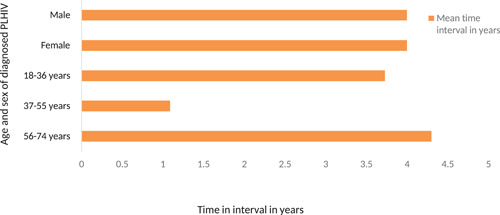
Mean HIV time interval between initial HIV infection and first diagnosis among naïve PLHIV from 1993 to November 2020, Bhutan

As shown in Figure 2, the mean time interval between the initial HIV infection till the first diagnosis was 4 years for both the male and female. There was not much mean time interval difference between the different age categories except among the aged 56–74 with 3.73 years unlike 4.4. and 4.09 among those aged 18–36 and 37–55 years, respectively.

**Figure 3:**
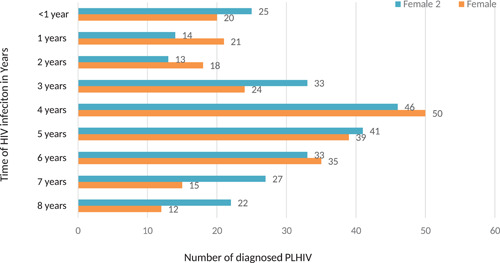
Time interval between the HIV infection till diagnosis among the naïve PLHIV in Bhutan from 1993 to June 2020

As depicted in Figure 3 below, 15.57% of the study population had a time interval of 7–8 years between initial infection and diagnosis. The majority 45.90% were found to have been infected between 5 and 8 years. While 25.2% were infected between 1 and 3 years and 9.22%% were diagnosed within less than 1 year of infection. Within the time range of 1–8 years majority (19.67%, *n* = 96) of the participants have acquired the HIV infection about four years back. Although there is not much difference in the time interval of HIV acquisition between males and females it was found that there are fewer females as compared to males whose initial infection dated back to 7 and 8 years, respectively. The decreasing trend has been observed in both males and females whose initial HIV infection accounted to 5–8 years but it was found statistically not significant.

## 4 DISCUSSION

In our study, we had slightly more male clients than female. The median age was 32 years. The overall distribution of CD4 count by sex revealed that about 45% of the participants had CD4 count cell less than 350 cells/ml and the frequency distribution also showed 50th percentile of participants having CD4 count below 325 cells/ml which corroborates to WHO clinical Stage 3 and 4 of HIV infection indicating advanced and severe immunosuppression.^7^ This shows the late diagnosis of HIV in Bhutan. This also shows that most of the infected individuals remains unaware of their HIV status until they witnessed advanced disease with severe symptoms and visited the health facility. This is evident from the current reported HIV cases in Bhutan where diagnosis through medical screening is the second highest (28%) among all entry points.^1^ This is also consistent with several other studies where the majority of the PLHIV were diagnosed late due to many factors such as poor knowledge of the importance of testing and treatment, inaccessibility to HIV prevention services, stigma, and discriminations.^25^, ^26^

Our findings also showed that females reported slightly higher CD4 count as compared to males although the differences were not statistically significant. Meaning any gender if delayed in testing can lead to a low CD4 count at the point in time provided other factors do not hinder the overall body immune system. This is in agreement with findings from other studies which have also recorded a higher CD4 count among females than in males.^27^, ^28^, ^29^ One reason could be due to early initiation of HIV testing in women due to antenatal checkup which validates with the national epidemiological data where the majority of the reported cases belongs to housewives.^1^ Bhutan's policy states two times (first trimester and third trimester) HIV testing for all the pregnant mothers to ensure zero vertical transmission to gear towards triple elimination of mother‐to‐child transmission of HIV, Hepatitis B, and Syphilis. Furthermore, the sex hormones are also suggested to influences the CD4 count in the human body where women are more likely to have a higher CD4 count as compared with men.^30^, ^31^

The study also revealed that only 20% of PLHIV had timely diagnosis when they were at clinical Stage 1 with no signs and symptoms of AIDS‐related illness. The evidence across the globe shows that early diagnosis is important to initiate timely treatment for quality life and then prevent onward transmission of HIV from the source.^32^ Therefore, routine HIV testing is a cost‐effective measure than to delay testing and progress into advanced disease. The study by Branson et al. showed cost‐effectiveness of routine HIV testing at less than USD 50,000 per QALY gained for a prevalence of undiagnosed HIV infection as low as 0.2%.^33^ Therefore, the current new strategy of provider‐initiated HIV testing by the Ministry of Health in all the outpatient department (OPD) and inpatient Departments (IPD) besides other testing sites is justifiable for primary HIV case detection. The evidence shows that screening for HIV infection among those visiting patients with flu‐like symptoms to detect primary infection is important. This evidence has proven consistent with Bhutan's experience of diagnosing HIV cases from the 54 walk‐in flu clinics established as a part of the national COVID‐19 response in the country.^34^

Distribution of CD4 count by age showed that the youngest age category (18–36 years) had the highest CD4 count across the different CD4 categories. This indicates that HIV infection in Bhutan is concentrated among the economically reproductive age group which is in agreement with the current reported cases where the majority (87%) of the reported cases are between the ages of 15 and 49 years.^1^ Unlike other age categories the distribution of CD4 count among the population aged 37–55 years showed the decreasing trend with the highest number of PLHIV having CD4 less than 200 cells/ml. This shows that participants between 37 and 55 years are most likely to delay in testing as compared with other age categories. This is evident from the finding of this study where a greater number of PLHIV aged (18–36 and 37–55) years having lower CD4 count falling within the WHO clinical Stage 3 and 4 diseases.

The study also revealed a similar mean time interval from the initial HIV infection to the first diagnosis of 4 years for both males and females. The mean time interval between the three age categories is also almost the same with a little less time among participants between 56 and 74 years as compared with two other lower age categories. This shows that the older population is testing earlier as compared with the younger population but it contradicts with findings of many other studies claiming older people tend to test HIV late as compared with younger individuals.^35^, ^36^ It can also be due to a smaller number of samples among the older age category. Although the majority of the participants have shown both (mean and absolute number of the time interval) of 4 years, about 45% of the cases have infected before 5–8 years which corresponded to a CD4 count below 350 cells/ml. This finding supports the earlier observation of substantial delay in testing by the Bhutanese populations. Therefore, it is important to consider advocating and educating early prevention and testing among all the categories of the population. The evidence has shown that early diagnosis is key to successful treatment of HIV that can have a quality life for the PLHIV and prevents onwards transmission.^37^ Therefore, besides facility‐based testing, the need to have community‐based HIV prevention including laymen testing using oral swap HIV self‐testing to enhance early diagnosis among the key and vulnerable populations is critical in ending the AIDS epidemic by 2030. The effectiveness of such strategies is well‐proven across the globe.^38^, ^39^, ^40^

## 5 CONCLUSIONS

Our study showed the mean CD4 was 345 cells/ml for both males and females. The time interval from the initial HIV infection till the first diagnosis was 4 years. The study also revealed that about 45.90% with an almost equal proportion of males and females had a time interval of 5–8 years from the initial HIV infection to the first diagnosis. While 25.2% were infected between 1 and 3 years and 9.22%% were diagnosed within less than 1 year of infection. The majority of the clients were between the age of 18 and 55 years and CD4 count between 200 and 499 cells/ml. Despite the ongoing high level advocacy, public health education, and extensive voluntary counseling and testing (VCT), the study has revealed that a large number of people did not present until the advanced disease. Therefore, we recommend strengthening medical screening, community‐based HIV prevention, and HIV self‐testing to enhance early case diagnosis and treatment besides the facility‐based and standalone VCT/HISC centers.

## CONFLICT OF INTERESTS

The authors declare that there are no conflict of interests.

## AUTHOR CONTRIBUTIONS

Lekey khandu conceived the research questions, developed the methods used, analyzed the data, interpreted results, and drafted the manuscript. Guru P. Dhakal contributed to the study design, protocol development, interpretation of data, and reviewed the manuscript. Karma Lhazeen contributed to the conception, acquisition or interpretation of data; critical review of the content.

## Data Availability

The data that support the findings of this study are available on request from the corresponding author. The data are not publicly available due to privacy or ethical restrictions.
